# 含艾伏尼布方案治疗IDH1突变急性髓系白血病16例的疗效和安全性分析

**DOI:** 10.3760/cma.j.cn121090-20251125-00546

**Published:** 2026-07

**Authors:** 丹丹 陈, 嘉成 徐, 昭 尹, 鹏程 史, 丹 徐, 洁瑜 叶, 慧 刘, 丽 宣, 钰 张, 启发 刘, 国攀 余

**Affiliations:** 1 南方医科大学南方医院血液科，广州 510515 Department of Hematology, Nanfang Hospital, Southern Medical University, Guangzhou 510515, China; 2 暨南大学附属广东省第二人民医院血液科，广州 510317 Department of Hematology, Guangdong Second Provincial General Hospital, Jinan University, Guangzhou 510317, China

## Abstract

为评估真实世界中艾伏尼布（Ivosidenib，IVO）治疗IDH1突变急性髓系白血病（AML）患者的疗效和安全性，回顾性分析2021年9月至2025年5月南方医科大学南方医院收治的16例接受IVO治疗的IDH1突变AML患者临床资料。患者中位年龄57（25～80）岁，男7例，女9例，6例（37.5％）患者的ECOG评分为3～4分。14例（87.5％）为欧洲白血病网（ELN）危险分层中高危组。5例（31.2％）接受VA（维奈克拉+阿扎胞苷）联合IVO诱导治疗，3例获完全缓解（CR），其中2例可检测残留病（MRD）转阴并伴有IDH1数字PCR（dPCR）阴性；2例（12.5％）接受VA+IVO巩固治疗，均维持CR，其中1例MRD阴性且伴IDH1 dPCR阴性；4例（25.0％）复发/难治患者接受维奈克拉+去甲基化药物+IVO（3例）或IVO单药（1例）挽救治疗，均获CR，其中3例MRD阴性，1例伴IDH1 dPCR阴性；5例（31.2％）接受IVO维持治疗，持续缓解时间（DOR）>12个月。主要≥3级不良事件为中性粒细胞减少（56.3％）、贫血（43.8％）及血小板减少（37.5％），IVO相关不良反应包括分化综合征（12.5％）及QT间期延长（12.5％），无患者死于治疗相关并发症。提示含IVO方案用于IDH1突变AML各阶段治疗显示良好疗效与安全性，值得临床进一步推广运用。

## Abstract (en)

To evaluate the real-world application of ivosidenib（IVO）in patients with IDH1-mutated acute myeloid leukemia（AML）, as well as its efficacy and safety. We retrospectively analyzed clinical data from 16 patients with IDH1-mutated AML who received IVO-based therapy at Nanfang Hospital between September 2021 and May 2025. The median age was 57 years（range, 25–80 years）, with 7 males and 9 females. Six patients（37.5％）had an Eastern Cooperative Oncology Group performance status of 3–4, and 14 patients（87.5％）were classified as intermediate- or high-risk according to the European LeukemiaNet criteria. Among 5 patients（31.2％）receiving venetoclax（VEN）plus azacitidine（AZA）combined with IVO for induction, 3 achieved complete remission（CR）, including 2 with measurable residual disease（MRD）-negative and IDH1 dPCR-negative status. Two patients（12.5％）received VA plus IVO for consolidation; both maintained CR, with 1 achieving MRD-negative and IDH1 dPCR-negative status. Four relapsed or refractory patients（25.0％）received VEN plus hypomethylating agents（HMA）combined with IVO（*n*＝3）or IVO alone（*n*＝1）for salvage therapy. All patients achieved CR, of whom 3 obtained MRD-negative results, and 1 was IDH1 dPCR-negative. Five patients（31.2％）received IVO as maintenance therapy, with a duration of response exceeding 12 months. The most common grade ≥3 adverse events were neutropenia（56.3％）, anemia（43.8％）, and thrombocytopenia（37.5％）. IVO-related adverse events included differentiation syndrome（12.5％）and QT interval prolongation（12.5％）, and no treatment-related deaths were observed. IVO-based regimens demonstrated favorable efficacy and a manageable safety profile across all treatment phases in patients with IDH1-mutated AML, supporting their broader clinical application.

急性髓系白血病（AML）是一类高度异质性的恶性血液系统肿瘤，疾病复发仍是治疗失败的主要原因[1]–[3]。异柠檬酸脱氢酶1（IDH1）基因突变是AML重要的分子异常之一，并已成为重要的靶向治疗标志物[4]–[5]。艾伏尼布（Ivosidenib，IVO）作为一种选择性IDH1抑制剂，目前已被国内外指南推荐用于新诊断不适合强化化疗或复发难治性IDH1突变AML患者的治疗[6]–[7]。多中心研究结果显示，阿扎胞苷（AZA）联合IVO一线治疗不适合强化化疗IDH1突变AML患者的完全缓解（CR）/部分血细胞恢复的完全缓解（CRh）率为52.8％[8]。IVO单药治疗复发难治患者的CR/CRh率为36.7％[9]。另外，IVO被用于异基因造血干细胞移植（allo-HSCT）后的维持治疗同样具有良好的安全性与疗效[10]。但是，由于可及性问题，中国真实世界中使用IVO治疗AML的数据仍较少。因此，本研究拟对单中心接受含IVO方案治疗的IDH1突变AML患者的临床资料进行回顾性分析，进一步评估含IVO方案用药的场景，以及临床疗效与安全性，为临床用药提供参考。

## 病例与方法

一、病例

本研究纳入2021年9月1日至2025年5月31日南方医科大学南方医院收治的IDH1突变AML患者。所有患者均符合WHO 2022诊断标准，并接受IVO治疗（单药或联合方案）至少1个疗程并完成疗效评估。IVO按标准剂量（500 mg，每日1次）连续给药，联合治疗组至少14 d，单药治疗组至少28 d；治疗期间根据合并用药（如使用三唑类药物）按照说明书进行IVO剂量调整，或因病情变化需要短暂中断IVO治疗，不作为排除标准。本研究排除同期接受其他精准靶向治疗（如FLT3抑制剂）或缺乏疗效评估的患者。

二、检测方法

1. 一代测序：采用Sanger测序技术对包括IDH1在内目标基因特定位点进行检测。经PCR扩增并纯化后的产物进行双向测序，测序结果经专业软件分析并与参考序列比对，用于鉴定单核苷酸变异及小片段插入或缺失。

2. 二代测序（NGS）：采用高通量测序技术对样本进行173个白血病相关基因的检测。测序在Illumina（HiSeq X、NovaSeq、MiSeq、MiniSeq、NextSeq）或华大智造（MGISEQ、DNBSEQ）平台上完成。原始数据经质量控制后，利用标准生物信息学流程（如GATK、MuTect2等）进行比对和变异分析，获得各靶基因的突变信息。

3. IDH1克隆清除评估：采用数字PCR（dPCR）检测IDH1突变克隆负荷，灵敏度可达0.001％，用于评估分子学清除。双阴定义：MRD阴性且IDH1 dPCR阴性，用于判定深度缓解。

三、疗效及安全性评估

患者的疗效评价基于IVO暴露期间或治疗后获得的最佳反应。疗效评估依据2025年欧洲白血病网（ELN）指南标准，包括CR、血细胞未完全恢复的完全缓解（CRi）、CRh、形态学无白血病状态（MLFS）、部分缓解（PR）及未缓解（NR）。复合完全缓解（CRc）率定义为CR率、CRi率与CRh率之和；总体反应率（ORR）为CRc率、MLFS率及PR率之和。流式细胞术检测MRD，MRD水平<0.1％定义为阴性[11]。总生存（OS）期定义为自确诊起至末次随访或因任何原因死亡的时间；无事件生存（EFS）期定义为自治疗开始至复发、死亡或末次随访的时间。对无治疗反应者，EFS期记为1 d。

不良反应依据美国国家癌症研究所常见毒性分级标准（NCI CTCAE5.0版）进行分级与评估。出现不良反应时，根据具体情况给予相应对症及支持治疗，并密切监测其转归情况。

四、随访

通过医院住院及门诊电子病历系统进行随访，随访截止时间为2025年7月30日。

五、统计学处理

采用SPSS 27.0和R 4.4.0软件进行统计分析。计量资料以中位数（范围）表示，计数资料以例数及百分比表示。组间差异采用*χ*²检验或Fisher确切概率法进行比较。生存分析采用Kaplan-Meier法，随访时间采用反向Kaplan-Meier法计算。以*P*<0.05为差异具有统计学意义。

## 结果

1. 临床特征：共有16例患者符合本研究纳入标准纳入分析，中位年龄为57（25～80）岁，男7例，女9例，其中6例（37.5％）的ECOG评分为3～4分。12例（75.0％）为原发AML，4例（25.0％）为继发/治疗相关性AML。根据ELN2022危险分层，2例（12.5％）为低危组，6例（37.5％）为中危组，8例（50.0％）为高危组。4例（25.0％）为复杂核型。最常见的伴随基因突变为N/KRAS（6例，37.5％），其他依次为DNMT3A（5例，31.3％）、ASXL1（4例，25.0％）、NPM1（3例，18.7％）、TET2（3例，18.7％）等。IDH1突变位点均位于R132，其中R132C最常见（7例，43.8％），其次为R132G（3例，18.7％）。

2. 治疗时机及治疗疗效：16例患者在不同治疗阶段接受IVO靶向治疗。5例（31.3％）在诱导阶段接受中位1（1～2）个疗程VA（维奈克拉+AZA）+IVO三药方案，其中3例均1个疗程获得CR，2例流式细胞术MRD和IDH1 dPCR均阴性。在巩固阶段，分别有1例（6.3％）接受2个疗程VA+IVO方案，1例（6.3％）接受3个疗程IVO+AZA方案，两例均获得MRD阴性，其中1例IDH1 dPCR阴性。在4例（25.0％）复发/难治性患者中，3例均接受1个疗程VA+IVO挽救治疗；另外1例患者VA方案治疗复发后接受IVO治疗45 d获得MLFS，维持半年后再次复发，再次接受1个疗程VD+IVO后获得CRh，4例患者均获得CRc，其中MRD阴性3例（75.0％），1例（25.0％）IDH1 dPCR阴性。另外有5例（31.3％）接受IVO单药维持治疗中位3（1～29）个月，均维持持续MRD阴性CR，且4例获得IDH1 dPCR阴性。具体治疗方案和疗效详见图1、表1。复杂核型与非复杂核型、不同IDH1突变亚型（R132C、R132G等）患者的疗效见表2。

**图1:**
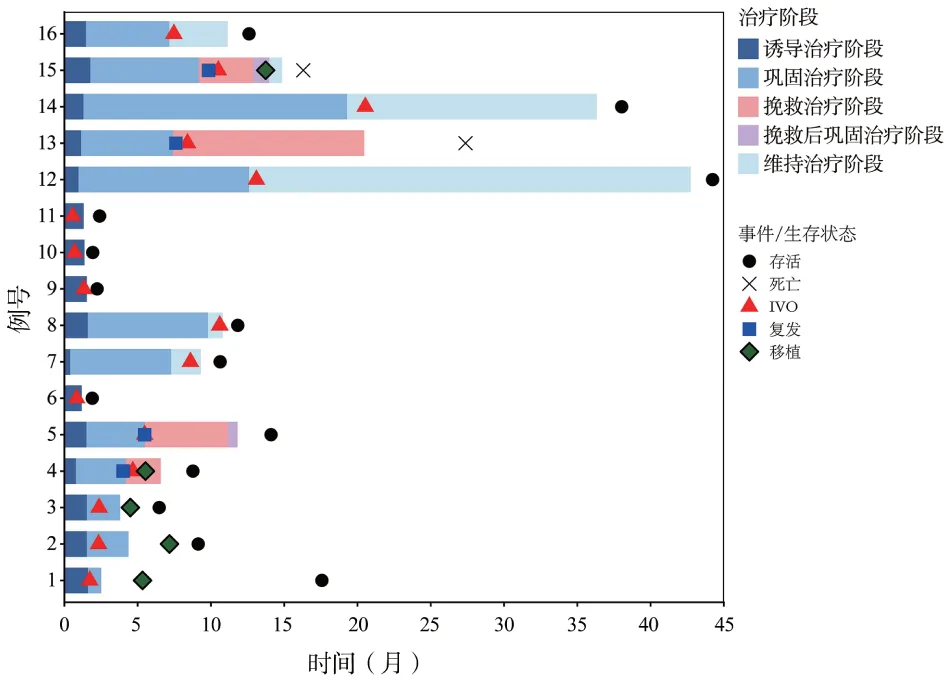
16例接受艾伏尼布（IVO）治疗的IDH1突变急性髓系白血病患者治疗泳道图

**表1:** 16例急性髓系白血病患者在不同治疗阶段接受艾伏尼布后疗效（例）

治疗阶段	例数	CRc	MRD阴性	IDH1阴性	IDH1 dPCR阴性	MRD+IDH1 dPCR双阴性
诱导期	5	3	2	2	2	2
巩固期	2	–	1	2	1	1
挽救期	4	4	3	1	1	1
维持期	5	–	5	4	4	4

**注** CRc：复合完全缓解；MRD：可检测残留病；IDH1 dPCR：数字PCR检测IDH1突变；–：不适用

**表2:** 不同细胞遗传学类型及IDH1突变位点患者的治疗反应［例（％）］

因素	例数	CRc	MRD阴性
细胞遗传学类型			
复杂核型	4	3（75.0）	3（75.0）
非复杂核型	12	11（91.6）	9（75.0）
IDH1突变位点			
R132C	7	6（85.7）	5（71.4）
R132G	3	3（100.0）	3（100.0）
R132H/L/S	6	5（83.3）	5（83.3）

**注** CRc：复合完全缓解；MRD：可检测残留病

3. 预后与转归：截至2025年7月30日，中位随访时间10.6（95％ *CI*：6.5～未达到）个月，6例（37.5％）桥接allo-HSCT，移植前均为CR/CRi。IVO治疗期间仅有1例（6.3％）患者出现复发；有2例（12.5％）死亡（1例患者死于疾病复发，1例患者死于allo-HSCT后合并噬血细胞综合征）。中位OS时间未达到，2年OS率和EFS率分别为（80.0±17.9）％和（59.5±16.2）％，3年OS率和EFS率分别为（53.3±24.8）％和（59.5±16.2）％（图2）。

**图2:**
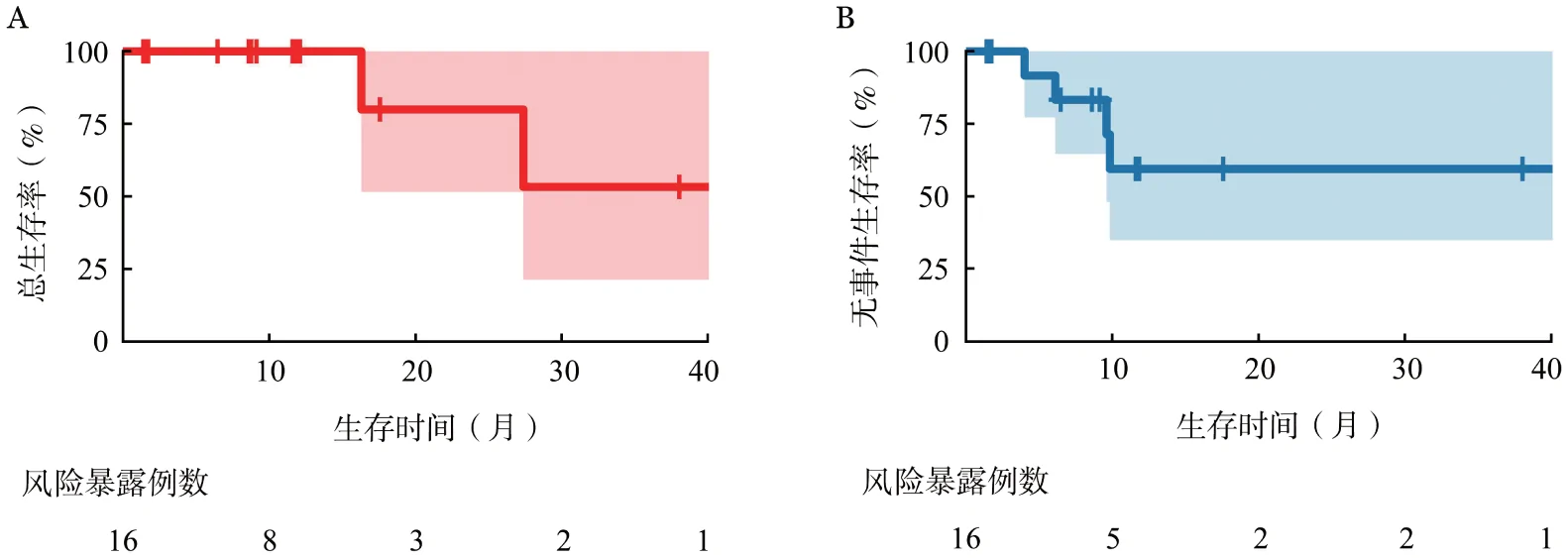
16例IDH1突变急性髓系白血病患者总生存（A）和无事件生存（B）曲线

4. 安全性评估：在血液学不良反应方面，有9例（56.3％）患者发生≥3级中性粒细胞减少，4例（25.0％％）出现≥3级粒细胞缺乏伴发热（FN）；7例（43.8％）发生贫血，其中5例（31.3％）≥3级；6例（37.5％）患者发生血小板减少，其中5例（31.3％）≥3级。在非血液学不良反应方面，6例（37.5％）发生肺部感染，其中2例（12.5％）≥3级；2例（12.5％）发生败血症，均≥3级；3例（18.8％）出现恶心呕吐，1例（6.3％）≥3级；1例（6.3％）出现过敏反应；2例（12.5％）出现分化综合征；2例（12.5％）出现QT间期延长，其中1例（6.3％）≥3级，1例（6.3％）患者被认为与治疗相关。

## 讨论

本研究回顾性总结单中心16例IDH1突变AML患者接受含IVO方案治疗的临床资料。结果显示，IVO可应用于诱导、巩固、维持及挽救治疗等不同阶段，并表现出良好的疗效和安全性。除维持治疗外，IVO多以联合方案应用，其中IVO联合VEN及去甲基化药物（VA方案）最为常见。既往研究表明，IVO+VA方案在初治及复发难治IDH1突变AML患者中可获得较高的缓解率及深度分子学缓解[10]，[12]–[14]。本研究中，接受该方案治疗的患者同样取得较好的疗效：一线治疗5例，3例获得CRc，其中2例实现MRD阴性及IDH1克隆清除；巩固治疗患者获得持续MRD阴性缓解。4例挽救治疗患者均获得CRc，3例达到MRD阴性，1例实现IDH1清除，随访期间未出现复发。与既往IVO联合AZA治疗或IVO单药治疗研究相比[15]–[16]，本研究观察到缓解率呈提高趋势，提示IVO+VA三药方案可能具有更强的抗白血病活性。此外，本研究中5例患者在缓解维持阶段持续接受IVO治疗，其中4例获得IDH1克隆清除，仅1例出现复发，且总体耐受性良好，提示IVO维持治疗可能有助于降低IDH1突变AML患者的复发风险，这与既往报道基本一致[17]–[18]。然而，鉴于本研究样本量较小、不同治疗阶段患者数量有限，上述结果仍需在更大样本的前瞻性研究中进一步验证。

本研究同时采用流式细胞术检测MRD及dPCR定量检测IDH1方法对疾病缓解深度进行评估。两种检测结果阴性预测整体一致：诱导和巩固治疗阶段二者阴性结果100％一致；挽救和维持治疗阶段，流式细胞术MRD阴性患者中仅部分IDH1 dPCR阴性，而MRD阳性患者全部IDH1 dPCR阳性，挽救组阴性一致率为1/3，维持组为4/5。上述差异可能与两种检测方法灵敏度不同有关，流式细胞术MRD检测界值约为0.1％，而dPCR可达0.001％。以上结果支持dPCR检测IDH1克隆清除可作为IDH1突变AML深度MRD检测的有效方法，这与文献报道一致[19]–[20]。

从机制上看，IDH1突变通过产生2-HG导致表观遗传异常及分化阻滞，而IVO可通过抑制突变型IDH1酶活性促进白血病细胞分化[10],[21]。同时，IDH1/2突变AML细胞对BCL-2介导的凋亡途径具有较高依赖性，因此IVO与VEN联合具有协同抗白血病作用[12],[22]。值得注意的是，本研究中5例接受VA+IVO诱导治疗的患者中仍有2例未获得CR。既往研究提示，共突变谱差异、获得性耐药以及抗凋亡依赖改变等因素均可能影响治疗反应[12],[23]–[27]。本研究两例未获缓解患者分别伴TP53突变和IDH2共突变，这些因素可能部分解释其疗效不佳。

在安全性方面，本研究中最常见的不良事件为中性粒细胞减少（56.3％）、贫血（43.8％）及血小板减少（37.5％），非血液学不良事件以感染（37.5％）为主；少数患者（12.5％）出现分化综合征及QT间期延长。总体来看，IVO单药及其联合低强度化疗方案耐受性良好，未出现新的或未预期毒性，与既往研究报道基本一致[8],[28]–[29]。

本研究的主要局限性在于单中心回顾性设计及样本量较小，不同治疗阶段病例数有限，且随访时间较短，限制了对长期生存获益及亚组差异的分析。因此仍需多中心、大样本前瞻性研究进一步验证IVO在不同治疗阶段的最佳应用策略，并探索其耐药机制与优化联合方案。

综上，IVO在IDH1突变AML不同治疗阶段中均显示出良好疗效与安全性，尤其VA+IVO三药方案具潜在治疗优势，为进一步优化AML个体化精准治疗提供依据。
